# Bisulfite-Converted DNA Quantity Evaluation: A Multiplex Quantitative Real-Time PCR System for Evaluation of Bisulfite Conversion

**DOI:** 10.3389/fgene.2021.618955

**Published:** 2021-02-25

**Authors:** Sae Rom Hong, Kyoung-Jin Shin

**Affiliations:** Department of Forensic Medicine and Brain Korea 21 PLUS Project for Medical Science, Yonsei University College of Medicine, Seoul, South Korea

**Keywords:** bisulfite conversion, cytosine-free primer, DNA methylation, quantitative real-time PCR, TaqMan probe

## Abstract

Bisulfite (BS) conversion, which includes a series of chemical reactions using bisulfite, is a prerequisite to most DNA methylation analysis methods, and thus is an essential step in the associated research process. Unfortunately, BS conversion leads to the degradation or loss of DNA, which can hinder further downstream analysis. In addition, it is well known that incomplete BS conversion is crucial, as it causes an exaggeration of the DNA methylation level, which can adversely affect the results. Therefore, there have been many attempts to measure three key features of BS conversion: BS conversion efficiency, recovery, and degradation level. In this study, a multiplex quantitative real-time PCR system named BisQuE was suggested to simultaneously analyze three important aspects of the conversion step. By adopting cytosine-free PCR primers for two differently sized multicopy regions, the short amplicon and long amplicon were obtained from both the genomic and BS-converted DNA, thus enabling the obtaining of reliable and sensitive results and the calculation of the degradation level of the conversion step. Also, probes for detecting converted/unconverted templates and C-T indicators for inducing the formula were included in this assay to quantify BS-converted DNA in order to compute the conversion efficiency and recovery. Six BS conversion kits (EZ DNA Methylation-Lightning Kit, Premium Bisulfite kit, MethylEdge^®^ Bisulfite Conversion System, EpiJET Bisulfite Conversion Kit, EpiTect Fast DNA Bisulfite Kit, and NEBNext^®^ Enzymatic Methyl-seq Conversion Module) were tested in 20 samples using 50 ng of genomic DNA as an input with the BisQuE. The conversion efficiency, degradation levels, as well as recovery rates of the kits were investigated. A total of 99.61–99.90% conversion efficiency was perceived for five of the kits, while the NEBNext kit showed about 94%. The lowest degradation level was shown by the NEBNext kit, whereas the other kits were quite similar. The recovery rates of the kits were found to be within the range of 18–50%. A Qubit assay was also used to compare the recovery rate of BisQuE.

## Introduction

DNA methylation (DNAm) is one of the best-studied epigenetic phenomena for aging (Bell et al., 2019), development (Cedar and Bergman, 2009), and diseases (Greenberg and Bourc’his, 2019). Given that DNAm has potential implications in various fields, several DNAm analysis methods are available nowadays, including pyrosequencing, high resolution melting analysis, and massively parallel sequencing (MPS) (Kurdyukov and Bullock, 2016; Vidaki and Kayser, 2018). Bisulfite (BS) conversion, which includes a series of chemical reactions using bisulfite, is a prerequisite to most of the methods; therefore, it is considered to be an essential step in the associated research process. Unfortunately, BS conversion occurs under harsh conditions (acidic and high temperature) and thus leads to the degradation or loss of DNA, which can hinder further downstream analysis (Grunau et al., 2001; Tanaka and Okamoto, 2007; Holmes et al., 2014). In addition, incomplete BS conversion can have crucial implications, since it causes an exaggeration of the DNAm level, which might affect the results. For these reasons, many researchers have attempted to quantify BS conversion efficiency (Bryzgunova, 2013; Izzi et al., 2014; Leontiou et al., 2015; Worm Ørntoft et al., 2017; Kint et al., 2018; Tierling et al., 2018; Heidegger et al., 2020), recovery (Holmes et al., 2014; Leontiou et al., 2015; Worm Ørntoft et al., 2017; Kint et al., 2018; Tierling et al., 2018), and the degradation level (Grunau et al., 2001; Holmes et al., 2014; Kint et al., 2018).

Bryzgunova (2013) suggested the use of the quantitative real-time PCR (qPCR) of L1RE1 for obtaining the recovery rate and pyrosequencing for acquiring BS conversion efficacy; however, this work should be done separately. Izzi et al. (2014) pyrosequenced four genes to investigate the C to T ratio for assessing BS conversion efficiency. Holmes et al. (2014) measured the conversion efficiency of BS-converted DNA (BS-DNA) by Sanger sequencing of two different PCR products amplified with cytosine-free (Cfree) primers. Subsequently, UV spectrophotometer and qPCR based on two types of assays (methylation-specific and Cfree fragment) were used for quantifying the recovery rate along with further gel electrophoresis for the DNA degradation. This work provided performance tests for nine BS conversion kits and explained how the Cfree primer can be used. Leontiou et al. (2015) tested four commercial kits regarding BS conversion efficiency and recovery rate, Sanger sequencing and MPS for the efficiency, as well as fluorometer for the recovery. Low recovery was considered as fragmentation; however, fluorometric results only reflected the loss of DNA. Worm Ørntoft et al. (2017) aimed to compare the cell-free DNA recovery of 12 BS conversion kits, so they exploited several assays, including qPCR, droplet digital PCR (dPCR), MethyLight, and urea PAGE. Therefore, it can be concluded that it is hard to obtain the recovery and BS conversion efficiency of a single assay. Kint et al. (2018) used various sizes of amplicons for qPCR and dPCR to evaluate DNA fragmentation and recovery during the BS conversion step. In addition, they exploited MPS for calculating the conversion ratio, and measured the genomic DNA (gDNA) and ssDNA (BS-DNA) with Qubit. By dPCR, it was possible to analyze the DNA recovery and degradation at once; however, another step was needed to test the conversion rate.

DNA methylation is not only crucial for the detection of diseases, but it is also vigorously investigated in the field of forensic sciences, giving rise to forensic epigenetics (Parson, 2018; Vidaki and Kayser, 2018). In particular, age prediction based on the alteration of the methylome by aging and body fluid identification using methylations based on cell type is a widely researched area (Parson, 2018; Vidaki and Kayser, 2018). Therefore, the forensic applications of DNAm are expected to provide additional information from pieces of evidence due to its potential coverage, and support the identification of the origin of biological fluids left in crime scenes, enabling the inferring of crime acts. Also, an age estimation can narrow down the range of the unknown suspects. However, as the field of forensics has limitations in regard to the amount of obtainable DNA, the amount of available gDNA should be considered.

Tierling et al. (2018) studied the applicability of as little as 1 ng of BS-DNA and considered the oxidized forms of 5-methyl C. They visualized DNA recovery by gel electrophoresis of the PCR product of BS-DNA and calculated the conversion rate based on MPS data. Also, BS conversion kits were tested for BS conversion efficiency by MPS results and the degradation of kits by Qubit and Bioanalyzer results of 308 bp during a monoplex PCR had a minimum of 500 pg (Heidegger et al., 2020). Previous studies have attempted to develop many methods, which were shown to be highly accurate, although all of them had hardships with providing key information of the BS conversion: the BS conversion efficiency, recovery, and degradation level.

In this study, a multiplex quantitative real-time PCR system for the evaluation of BS conversion (BisQuE) was developed after simultaneously analyzing three important aspects of the conversion step. To suggest the degradation level of BS-DNA and reliability and sensitivity of the method, the concept of the Quantifiler Trio DNA Quantification kit (Thermo Fisher Scientific) was referred. This kit contains two different-sized multicopy targets (80 and 214-bp) on the human autosomal chromosome for degradation index to indicate whether the measured sample can be amplified to a full short tandem repeat profile or not. In the BisQuE, by adopting Cfree PCR primers for two different-sized multicopy regions, 104 bp of the short-sized amplicon and 238 bp of the long-sized amplicon were obtained from both the gDNA and the BS-DNA in a single assay. Furthermore, the designed probes to separate C and T on the non-CpG context of the short-sized amplicon can quantify a specific amount of the unconverted DNA (or gDNA) and converted DNA. Also, artificial internal positive control (IPC) and its PCR primers and probe were included in this assay to check the presence of PCR inhibitors in BS-DNA. For exploitation of the constructed qPCR system, six BS conversion kits were tested in 20 samples using 50 ng of gDNA as an input. The conversion efficiency, recovery, and degradation levels of the various kits were also investigated. For future studies, it is highly recommended to determine the BS conversion kit according to the purpose of the specific study. Moreover, acquiring the appropriate amount of PCR-functional DNA to obtain reliable data when the amount of input DNA is fairly low should be taken into account.

## Materials and Methods

### Sample and Bisulfite Conversion

Peripheral blood samples of 20 Korean subjects (10 males and 10 females) were obtained from a biobank of the Asian Sample Network under the approval of the Institutional Review Board of Severance Hospital, Yonsei University in Seoul, South Korea (4-2019-0707). DNA was extracted from a 200 μl whole blood sample using a QIAamp^®^ DNA Mini Kit (Qiagen, Hilden, Germany) following the instructions of the manufacturer. The extracted DNA was quantified using the Quantifiler^TM^ Duo Kit (Thermo Fisher Scientific, Waltham, MA, United States) and stored at −20°C until further use.

For the 20 samples, each sample was diluted to 5 ng/μl based on the Quantifiler Duo Kit result for the BS conversion and BisQuE step. The BS-DNA was obtained through the modification of 50 ng of gDNA, with six BS conversion kits (Supplementary Table S1): EZ DNA Methylation-Lightning Kit (Zymo Research, Irvine, CA, United States; Z-EZ), Premium Bisulfite kit (Diagenode, Seraing, Belgium; D-PB), MethylEdge^®^ Bisulfite Conversion System (Promega, Madison, WI, United States; P-ME), EpiJET Bisulfite Conversion Kit (Thermo Fisher Scientific; T-EJ), EpiTect Fast DNA Bisulfite Kit (Qiagen; Q-EF), and NEBNext^®^ Enzymatic Methyl-seq Conversion Module (New England Biolabs, Ipswich, MA, United States; N-NE). The BS conversion as well as subsequent purification steps were performed according to the protocol of the respective kit and the BS-DNA was eluted with 10 μl of 1 × TE buffer except for the N-NE, which was eluted with 20 μl of 1 × TE buffer. Converted DNA was stored at −20°C and used within 1–3 days. Besides, 1 μl of BS-DNA samples was measured with the Qubit^TM^ ssDNA Assay Kit (Thermo Fisher Scientific) twice using a Qubit^TM^ 4 Fluorometer from the same manufacturer.

### Selection of Markers and IPC

**FIGURE 1:**
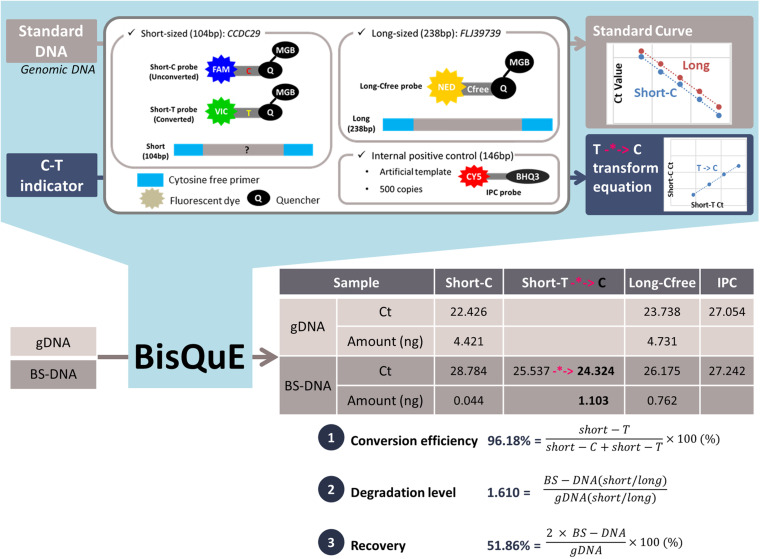
Concept of a multiplex quantitative real-time PCR evaluation system for bisulfite conversion (BisQuE) and an example. Genomic DNA (gDNA) and bisulfite-converted DNA (BS-DNA) undergoes the developed BisQuE method, including cytosine-free PCR primers and probes for two different-sized targets. Also, standard curves and the short-T to C transforming equation (-*->, highlighted in pink color) were obtained with standard DNA and C-T indicators, respectively. With the results of each gDNA and BS-DNA, the three key features (conversion efficiency, degradation level, and recovery) were calculated.

To achieve a measurement with high accuracy and sensitivity, multicopy regions that showed a relatively constant copy number among several populations (Sudmant et al., 2010) were considered. Based on *in silico* BS-converted genomic reference sequences, Cfree PCR primer candidates and probe candidates were designed with Primer3 v. 0.4.0^1^ and subsequently adjusted manually (Table 1). The region on introns of two genes, *CCDC29* (*ANKRD20A20P*) and *FLJ39739* were targeted to amplify 104 and 238 bp, respectively. Two types of *CCDC29* probes, the short-C probe and short-T probe were designed to separate BS-converted T and unconverted C on the same site in a non-CpG context (Figure 1). These probes had the same sequence except only one base: C for C probe and T for T probe (Table 1). Meanwhile, the probe of the longer amplicon, *FLJ39739*, included no cytosine nucleotide in its sequence, so that it could be detected both in gDNA and BS-DNA.

**TABLE 1:** Primer and probe sequences for qPCR.

	PCR amplification			Probe		
		
Target	Primer sequences (5′ > 3′)	Conc. (μM)	Amplicon size (bp)	Probe sequence (5′ > 3′)	Conc. (μM)	Length (nt)
Short	F: gaa atg gtt aag aga aag gga aa	0.6	104	C: FAM-tgg gtg aat a**C**t tag aat g-NFQ MGB	0.1	19
CCDC29* (*ANKRD20A20P*)	R: ccc att aca ttt ttc atc ctc a	0.6		T: VIC-tgg gtg aat a**T**t tag aat g-NFQ MGB	0.1	19
Long	F: ggg aaa atg agg aag tga tga	1.0	238	Cfree: NED-aat gtt gta tgt tat ttg tgg-NFQ MGB	0.15	21
FLJ39793**	R: aca caa aaa acc ctt caa aaa a	1.0				
IPC	F: aac tgc tag aaa acc gcg tc	0.8	147	Probe: CY5-tcc agg cag tgc gtc tgc tgt-BHQ3	0.2	21
	R: gag gca ggc tct tgc tat gt	0.8				

*chr9:67907355-67907458 and 8 other loci (GRCh38, UCSC in silico PCR).

**chr1:149482287 + 149482524 and 10 loci (GRCh38, UCSC in silico PCR).

An IPC sequence was generated with a Random DNA Sequence Generator (Supplementary Table S2)^2^ to obtain a random sequence free from human DNA to avoid interaction with other primers or probes. IPC was synthesized by gBlocks Gene Fragments (Integrated DNA Technologies, Coralville, IA, United States) and calculated to be 500 copies for each qPCR reaction.

### Standard DNA and C-T Indicators

Standard DNA samples were prepared from Quantifiler Duo DNA Standard by serial dilution with TE buffer: 10, 2 ng, 400, 80, and 16 pg per 1 μl. In addition, 1 ng/μl of a BS-converted, methylated sample (Epi-M) and 1 ng/μl of a BS-converted, unmethylated sample (Epi-U) were obtained from the EpiTect Control DNA and Control DNA Set (Qiagen), which were diluted according to the concentration given by the manufacturer and utilized as reference samples. C-T indicators (Supplementary Table S2) were synthesized by Macrogen (Seoul, South Korea) and 10^6^, 10^5^, 10^4^, and 10^3^ copies per μl were freshly prepared to obtain the relationship between C to T. As the indicator sequence contained a degenerate base Y, it supposed to consist of 50% of C and 50% of T for simple calculation (Supplementary Table S2).

### BisQuE

The BisQuE was performed with an Applied Biosystems 7500 Real-Time PCR System (Thermo Fisher Scientific) and the results were analyzed with the Applied Biosystems 7500 Real-Time PCR Software v2.3 from the same manufacturer. As most of the ready-made real-time PCR systems contain uracil N-glycosylase or uracil-DNA glycosylase to prevent carryover contamination, BS-DNA could not be amplified in this system. To analyze the features of BS-DNA, each reaction contained 5 μl of 2X Platinum^TM^ II Hot-Start PCR Master Mix (Thermo Fisher Scientific), 0.6–1.0 μM of each primer, 0.1–0.2 μM of each TaqMan probe (Thermo Fisher Scientific) (Table 1), 500 copies of IPC, 0.5 μl of 1/10 diluted ROX passive reference dye (Thermo Fisher Scientific), and 1 μl of the template: 16 pg/μl–10 ng/μl of five DNA standards; 1 ng/μl of Epi-M; 1 ng/μl of Epi-U; 5 ng/μl of gDNA; BS-DNA; and 10^3^–10^6^ copies/μl of four C-T indicators. The thermal cycling conditions were set to 94°C for 2 min, followed by 40 cycles at 94°C for 15 s and 60°C for 45 s. All the reactions were amplified in duplicate (Supplementary Figure S1).

Cycle threshold (Ct) values were determined using the automatic baseline algorithm. The slope of the standard plot regression line was used to calculate qPCR amplification efficiencies. The content of each 104 bp of the *CCDC29* and 238 bp of the *FLJ39739* amplicon was calculated from the Ct value of the C probe of the *CCDC29* (short-C) and the Cfree probe of the *FLJ39739* (long-Cfree), respectively. The T probe of the *CCDC29* (short-T) was subsequently computed from the relationship between the Ct values of the short-C and the short-T from the results of the C-T indicators.

The conversion efficiency, degradation level, and recovery of BS conversion were acquired based on the calculated content of each amplicon. The ratio of short-T amount and sum of the short-C and the short-T amount (%) described the BS conversion efficiency and the ratio of the division of the short by the long amplicon of gDNA and BS-DNA led to the calculation of the degradation level. Besides, double the short amplicon ratio of the gDNA and BS-DNA (%) was recovered from the BS conversion step (the recovery of the N-NE samples was doubled again as its eluted volume was 20 μl, whereas others were 10 μl) (Figure 1 and Supplementary Figure S2). These values were obtained for 120 BS-converted samples from 20 gDNA samples undergoing six different BS conversion kits.

### Statistics Analysis

To show if there were significant differences between BS conversion kits in each of conversion efficiency, degradation level, recovery from BisQuE, and recovery tested with Qubit. All statistical analysis was performed with IBM SPSS 25 and Microsoft Excel Office 365. A Shapiro-Wilk test was done to test normality of the three features and Qubit recovery, then a Levene’s test was done for testing equal variance. The following tests were applied by its results: Kruskal–Wallis one-way analysis of variance (ANOVA) for conversion efficiency; Welch’s one-way ANOVA for degradation level and Qubit recovery; one-way ANOVA for recovery from BisQuE. *Post hoc* analysis was performed to confirm the differences between the kits: Bonferroni-corrected method for conversion efficiency; Games-Howell test for degradation level and Qubit recovery; Tukey’s honest significant difference test for BisQuE recovery.

## Results

### Standard DNA, C-T Indicator, and Commercial BS-DNA

Data generated for the standard DNA showed a consistent assay sensitivity and reproducibility (Supplementary Table S3). Among five individual assays, 10 ng–16 pg of standard per well exhibited an average Ct of 21.247–29.899 in short-C and 22.595–31.077 in long-Cfree, respectively. Consistency of the results and assay reproducibility were also exhibited in high R-squared values, and the PCR efficiency, slope, and y-intercept were constant. With this result, standard curves of short-C and long-Cfree were obtained for each assay and then applied to quantify the amount of short amplicons containing cytosine (gDNA or unconverted DNA) and long amplicons. C-T indicator data also demonstrated similar results for the standard DNA (Supplementary Table S4) in terms of Ct values of each successive dilution, R-squared value, and PCR efficiency. The Ct transforming formula (highlighted in pink color in Figure 1) of short-T to short-C were generated with the relative ratio of Ct between short-C and short-T in four different amounts of diluted C-T indicators. The Ct value of the short-T of BS-DNA was substituted into the formula to convert the Ct of short-T into that of short-C, so that transformed Ct could be applied to the standard curve of short-C (Figure 1 and Supplementary Material 1). Meanwhile, 1 ng of the Epi-M and Epi-U samples were analyzed in common among the five assays, and their Ct values and calculated amount were similar in comparison with the standard DNA. Also, both of the two commercial samples showed a high average conversion rate of more than 99%. Those samples were found to have a much smaller amount (about 0.2 ng) than expected.

### Conversion Efficiency

**FIGURE 2:**
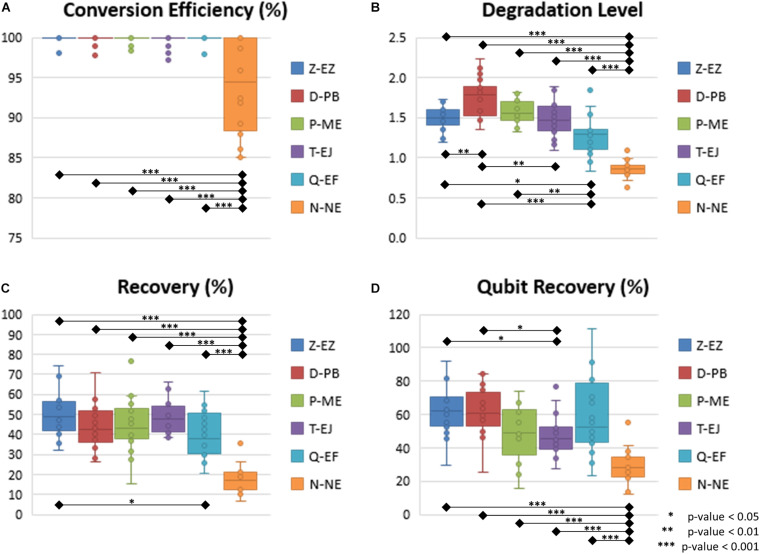
Box-scatter plot of BS conversion kit. **(A)** Conversion efficiency of each kit. **(B)** Degradation level of each kit. **(C)** Recovery calculated based on the qPCR data of each kit. **(D)** Recovery measured with the Qubit ssDNA assay. In all three key features of bisulfite conversion, N-NE showed the lowest performance, and it was significantly different to the other five kits.

The conversion efficiency of each kit was calculated by the short-T amount divided by the sum of the short-C and short-T amount as shown in Figure 1, Supplementary Figure S2, and Supplementary Material 1. Since standard DNA, which is human DNA, only provided the standard curves of short-C and long-Cfree, the Ct values of the short-T were modified into those of the short-C with the application of the relation formula obtained from the C-T indicator. As shown in Figure 2A, most of the BS-DNA exhibited more than 99% of conversion efficiency. In this study, Z-EZ was the highest efficiency in BS conversion, followed by the Q-EF, P-ME, D-PB, T-EJ, and N-NE. All samples converted with kits, except the N-NE, had a higher efficiency than 95%, but seven out of 20 samples from the N-NE showed less than 90% of the rate. In statistics analysis, there was a significant difference between N-NE and other kits (*p* < 0.001) and no significant differences were observed among the five kits.

### Degradation Level

Before computing the degradation level, the first step is to calculate the ratio of short amplicons (both short-C and short-T) divided by the long amplicons (long-Cfree). Degradation levels were computed from BS-DNA out of gDNA of this ratio (Figure 1, Supplementary Figure S2, and Supplementary Material 1). This concept is inspired from the degradation index of the quantifier HP and Trio DNA Quantification kit. Degradation level will be 1 when the ratio is the same in both gDNA and BS-DNA. However, if the amount of long fragment is smaller in the BS-DNA, the short/long ratio will be bigger leading to a degradation level of >1. Therefore, the degradation level >1 implies that the BS-DNA was degraded during the BS conversion step. Shown in Table 2 and Figure 2B, D-PB exhibited the highest degradation level and N-NE showed the lowest degradation level, less than 1.000, among the six tested kits. Obviously, N-NE was significantly different from the other five kits (Games-Howell’s *p* < 0.001). Other kits demonstrated average degradation levels ranging between 1.279 and 1.577. There was no difference between Z-EZ, P-ME, and T-EJ. Meanwhile, Q-EF showed significant difference with four kits including N-NE, Z-EZ (*p* < 0.05), D-PB (*p* < 0.001), and P-ME (*p* < 0.01). D-PB showed differences with Z-EZ and T-EJ (*p* < 0.01).

**TABLE 2:** Six BS conversion kits and an overview of the results.

No.	Abbreviation	Kit	DNA input (ng)	Elution vol. (μl)	qPCR						Qubit
	
					Conversion efficiency (%)	Conversion rank	Degradation level	DL rank	Recovery (%)	Recovery rank	Recovery (%)
1	Z-EZ	EZ DNA methylation-lightning kit	50	10	99.90	1	1.495	4	50.58	1	62.57
2	D-PB	Premium bisulfite kit		10	99.74	4	1.762	6	43.79	4	61.67
3	P-ME	MethylEdge bisulfite conversion system		10	99.78	3	1.577	5	44.42	3	49.12
4	T-EJ	EpiJET bisulfite conversion kit		10	99.61	5	1.479	3	48.39	2	47.12
5	Q-EF	EpiTect fast bisulfite kit		10	99.89	2	1.279	2	39.65	5	58.66
6	N-NE	NEBNext enzymatic methyl-seq conversion module		20*	94.24	6	0.857	1	18.24	6	27.90

*Due to the bead purification step, the converted DNA was eluted with 20 μl of TE buffer by following the manufacturers’ guide.

### Recovery From BisQuE and Qubit

In this study, the recovery of each kit was calculated by the ratio of the content of the short amplicon from BS-DNA (short-T and short-C, representing converted U, and unconverted C) divided by gDNA (short-C) and subsequently multiplied by two (Holmes et al., 2014; Figure 1, Supplementary Figure S2, and Supplementary Material 1). Because both the sense and antisense strand of the gDNA worked as template, only the sense strand of the BS-DNA could be amplified (Holmes et al., 2014). In addition, BS-DNA from the N-NE was eluted in 20 μl of TE buffer, so the recovery of the N-NE had to be multiplied by two again.

As shown in Table 2 and Figure 2C, Z-EZ exhibited the highest recovery and N-NE showed the lowest recovery, with 50.58 and 18.24%, respectively. The average recovery rates of the remaining kits, except for Q-EF, were highly similar ranging between 43.79–48.39%. In statistics analysis, there was a highly significant difference (*p* < 0.001) between N-NE and the other kits. Z-EZ and Q-EF showed significant difference (*p* < 0.05). However, the rest of each kit in this study was insignificantly different (Figure 2C).

Besides the usage of the developed qPCR system, the recovery was also measured with the Qubit ssDNA assay. As shown in Figure 2D, N-NE was significantly different to the other five kits (Games-Howell’s *p* < 0.001) and T-EJ showed significant difference with Z-EZ and D-PB (*p* < 0.05). However, there was no difference in the other kits. There was one sample that showed a recovery rate larger than 100% in the Q-EF kit, which showed 54.55% in the BisQuE result. Also, shown in Table 2 and Supplementary Figure S3, the low correlation between the qPCR result (Pearson’s rho = 0.446) as well as the rank by the Qubit assay and that by the qPCR were somewhat different.

## Discussion

### Overall

A BisQuE assay consisting of three sets of Cfree primers and four different probes (including IPC) was performed in this research (Figure 1 and Supplementary Figure S1). As the Cfree primers amplified both the BS-converted and unconverted DNA, the analysis of the gDNA and BS-DNA in a single assay was possible. Cfree primer systems were already exploited by Holmes et al. (2014); Worm Ørntoft et al. (2017), and Kint et al. (2018), but they were not able to obtain information regarding the BS conversion efficiency, degradation level, and recovery at the same time. More specifically, the extent of degradation of the BS-DNA can be provided by comparing the ratio of two different-sized amplicons from both the gDNA and the converted DNA. Furthermore, the short-C and the short-T probes were specific enough to separate converted T and unconverted C on non-CpG context. In addition, this assay aimed to amplify the multicopy region to achieve high sensitivity and reliability. Remarkably, standard DNA, which was from human genomic DNA, and gDNA had no Ct value in the short-T, and the short-C and short-T showed relatively constant patterns for the C-T indicator. On the other hand, the Ct value of IPC can indicate the presence of a PCR inhibitor, although there was no significant outlier Ct value of IPC in this research. IPC Ct values were found to be highly constant among samples (Supplementary Table S5), implying that there was an ineffective amount of PCR inhibitor in the 1 μl of the eluted DNA or buffering capacity of the used PCR master mix enabled to overcome it.

### C-T Indicator, Commercial BS-DNA, and gDNA

C-T indicator data showed high R-squared values among all the five BisQuE assays as shown in Supplementary Table S4. The C-T indicator is essential in this method. Since the standard curve of the short-T was not available, only indirect quantification with the standard curve of the short-C was possible by applying short-T Ct values to the transforming formula (Figure 1 and Supplementary Figure S2). If the R-squared value of the transforming formula was less than 0.99, the performed qPCR assay was unreliable. Therefore, the formula of the C-T indicator should be considered as important as the standard curves of the method. It is highly recommended to check the R-squared value of it and the PCR efficiency of C-T indicators. However, a C-T indicator contains a degenerate base Y and is supposed to consist of 50% of C and 50% of T for simple calculations in this study (Supplementary Table S2). As the Y in the middle of the sequence was synthesized, it is likely to have bias during the oligo synthesis process. Moreover, the purpose of the indicator was to induce the transforming formula for short-T to short-C to calculate the amount of converted DNA indirectly, not to separate them so they have the same Ct values. Therefore, this assumption of 50–50% would be acceptable.

Meanwhile, each 1 ng of Epi-M and Epi-U had a much smaller amount (about 0.2 ng) than expected; however, it suggested that even BS-converted samples that are available commercially might not meet the written concentration. Furthermore, a significantly higher short/long ratio was calculated for the Epi-U in comparison with the Epi-M. It is hard to conclude that the Epi-U was more degraded than the Epi-M due to its uncertain origin, as Epi-U samples originated from the cultured cell and it was impossible to test the original gDNA of the Epi-U. Therefore, before using commercial BS-DNA, quantitative measurement was a prerequisite to obtaining more reliable and accurate data in association with DNA methylation.

On the other hand, this qPCR system could also provide results for specific samples of gDNA. In this study, 5 ng of gDNA samples, which was quantified with Quantifiler Duo was measured. The average quantity calculated with short-C was 4.460 ng (1.141 ng of standard deviation) and that with long-Cfree was 3.981 ng (0.884 ng of standard deviation). This result is quite similar to the expectation: therefore, it might be possible to exploit the developed method to quantify gDNA as well as BS-DNA.

### Conversion Efficiency

All of the six kits used in this study showed high conversion efficiency and this result was concordant with previous studies. D-PB and P-ME were found to exhibit the highest conversion efficiency, more than 99%, by MPS (Leontiou et al., 2015). Similarly, high conversion rates of Q-EF were reported based on MPS results (Kint et al., 2018; Tierling et al., 2018) and Sanger sequencing data (Holmes et al., 2014). D-PB exhibited more than 99% of efficiency in MPS (Heidegger et al., 2020) and ddPCR (Worm Ørntoft et al., 2017), which also exhibited that T-EJ had a high conversion rate.

However, the efficiency of the Z-EZ was somewhat different in the studies. Tierling et al. (2018) suggested that Z-EZ unconverted 23% of cytosines in their study, but Holmes et al. (2014) and Kint et al. (2018) reported a high conversion efficiency of more than 99%. Izzi et al. (2014) showed that there was no significant difference between the Z-EZ and the EZ DNA Methylation Gold kit from the same manufacturer, which was reported to have the highest conversion rate (Holmes et al., 2014; Kint et al., 2018; Tierling et al., 2018). In our study, Z-EZ was observed to have the highest efficiency in BS conversion.

Since N-NE was quite recently introduced, it is less studied. To be precise, N-NE does not chemically alter C into U by using bisulfite in contrast with the other five kits, but it instead utilizes APOBEC for the enzymatic deamination of cytidine as well as bead purifications. Therefore, its performance might differ depending on the experience of the researchers or the given laboratory conditions.

It is widely known that incomplete BS conversion leads to exaggeration in the DNA methylation analysis, so choosing a BS conversion kit that guarantees high and stable conversion efficiency is one of the key steps in the preparation of a DNA methylation study. Five kits investigated in this study demonstrated extremely high conversion efficiency (average efficiency > 99.5%), whereas the other showed a high conversion rate (average > 94%). However, the application of the developed qPCR could be still useful before downstream experiments when it is necessary to confirm the success or failure of the BS conversion step due to mistakes of the researchers or mistreated reagents.

### Degradation Level

In this study, the short/long ratio only provided a glimpse of severe degradation; because the content of the short amplicon would be much higher than that of the long amplicon if there was severe degradation in association with the converted DNA. But to be precise, the copy number ratio of short and long amplicons can vary from individual to individual, the accurate determination of the quantity of degraded BS-DNA might be vague. Therefore, degradation level of BisQuE needed the simultaneous analysis of both the gDNA and the BS-DNA, so that it can normalize the individual copy number variation and enable the acquisition of a precise degradation level. Therefore, a degradation level >1 implies that the BS-DNA was degraded during the BS conversion step.

As shown in Table 2 and Figure 2B, the degradation level of the N-NE was lower than 1, meaning that the proportion of the long amplicons increased during the process. As mentioned in the section “Conversion Efficiency,” the BS-DNA from the N-NE was purified by beads; therefore, these findings might result from the bead purification step as the concentration of the beads contributed to the selective elimination of a specific size of DNA. A lower concentration of the beads led to a larger size of purified DNA, 1.0 × bead purification during the conversion could remove some of the small-sized DNA. The method using a larger volume of the bead, such as 2.0× or 3.0×, or the standard column purification method might enable the recovery of the short-fragments of enzyme-treated DNA more than the manufacturer’s guide. However, this study was the first to measure the conversion efficiency, degradation level, and recovery of N-NE, so it was hard to modify the guide. The suggested methods of the cleanup step would be recommended after testing the three features of the kit.

Other kits demonstrated average degradation levels ranging between 1.279 and 1.577. Kint et al. (2018) reported a similar result in the fragmentation of three kits: Q-EF, Z-EZ, and D-PB in a less degraded order. They compared the degradation of each kit with gel electrophoresis, qPCR, and dPCR. In particular, during the comparison of the absolute quantification dPCR results of pre- and post-BS conversion samples, they found that BS-treated DNA had lost about 97% of the fragments longer than 227 bp, which observation was quite similar to that of this study. Additionally, gel electrophoresis data (Holmes et al., 2014) and Bioanalyzer data (Tierling et al., 2018) presented that the Q-EF was less fragmented than the Z-EZ on a longer scale (>500 bp). On the other hand, Worm Ørntoft et al. (2017) reported that T-EJ and D-PB were the least fragmented kits, but T-EJ was slightly less degraded than D-PB, which was in concordance with the findings of this research. However, Q-EF showed larger variance even in the case of its lower degradation level; therefore, those instabilities should be considered by the researchers.

On the other hand, larger target sizes, such as 500 bp, are not guaranteed by this system as the size of the long amplicon is 237 bp > 238 bp. The BS conversion step is well known for degradation and as one of the impeding steps of amplifying the large size of genes. Therefore, if the interested region is quite large, for example it is more than 500 bp, the degradation level can be confirmed with other methods, such as gel electrophoresis (2 μg of gDNA and BS-DNA; Holmes et al., 2014) or Bioanalyzer (500 ng of gDNA for starting material; Tierling et al., 2018). In this study, only 50 ng of gDNA was used as the starting material in the BS step and eluted with 10 μl (20 μl for N-NE) of TE buffer. Therefore, methods using gel electrophoresis and Bioanalyzer were not suitable to direct confirmation. But our preliminary test result (Supplementary Figure S4) using the Agilent DNA 1000 kit with Bioanalyzer showed that the large amplicon (*FLJ39739*) was obviously less amplified than the short amplicon (*CCDC29*) in BS-DNA. So, it is highly recommended for studying larger sized targets to check the degradation level with other methods and start with a sufficient amount of DNA. Furthermore, the amplification of small-sized amplicons that are less than 300 bp is common in forensic genetics. This small amplicon strategy can be suited to the BisQuE.

Moreover, the degradation level can be helpful to analyze the DNAm of formalin-fixed, paraffin-embedded samples or degraded forensic samples by exposing them to a harsh environment: to adjust the amount of input DNA considering the amplicon size of the target markers, to interpret negative results, or to exclude pseudo-positive results. An extremely high degradation level of the BS-DNA sample implies the high possibility of failure to amplify the large size of the target amplicon, and the trustworthiness of the result of the downstream analysis will be uncertain. Consequently, the degradation level can offer a minimum guideline for obtaining reliable data during the DNAm analysis regarding the interpretation of challenging samples.

### Recovery

In this study, the recovery of each kit was calculated by the ratio of the short amplicon content of the gDNA and BS-DNA. Concerning DNAm studies, such as forensic fields studies that are inevitably required to deal with a limited amount of input DNA, an insufficient amount of BS-DNA can lead to a pitfall called the stochastic effect, therefore a certain amount of BS-DNA depending on the purpose of the study must be present to obtain reliable DNA methylation data (Naue et al., 2018). Therefore, the recovery of the BS conversion kit should be thoroughly investigated to determine the minimum input of gDNA for conversion.

As shown in Table 2 and Figure 2C, N-NE showed the lowest recovery (18.24%). Since the N-NE requires bead purification twice during the conversion process, a severe DNA loss can occur, the extent of which depends on the skillfulness of the researchers. For the remaining kits, except for the Q-EF, average recoveries were highly similar, ranging between 43.79 and 48.39%. Like these results, Holmes et al. (2014) reported a higher recovery of Z-EZ than Q-EF using qPCR, and Worm Ørntoft et al. (2017) investigated the recovery by qPCR and found a slightly higher recovery rate for the T-EJ compared to that of the D-PB. Although the order of recovery was somewhat different, the average recovery of each kit in this study was relatively similar to each other, except for the N-NE (Figure 2 C). Besides, the qPCR amplicon sizes varied for each study; therefore, it is recommended to apply the method, which targets the appropriate size.

The Qubit assay was used to obtain the recovery of BS-treated DNA (Leontiou et al., 2015; Kint et al., 2018). Leontiou et al. (2015) reported that the D-PB had the highest recovery, followed by the P-ME, and both were observed to be about 55%. Furthermore, Kint et al. (2018) showed the recovery rank by Qubit measurement to be the highest for D-PB, followed by Z-EZ, and Q-EF. In this study, Z-EZ was the highest, followed by D-PB and Q-EF in order, showing > 55% of recovery measured by Qubit. There was a low correlation between the results of the qPCR and the Qubit assay (Table 2, Figure 2D, and Supplementary Figure S3). Moreover, one sample converted with Q-EF showed more than 100% of Qubit recovery though it showed a 54.55% recovery rate in BisQuE. This might be due to the fact that the constructed qPCR system quantifies functional (amplifiable) DNA, whereas the Qubit assay measures fluorescent dye signal intercalating ssDNA specifically. If the amount provided by the Qubit assay is low, it could imply that the amount of BS-DNA quantified by the BisQuE is low. However, it cannot guarantee the amount of amplifiable converted DNA even though the results of the Qubit seem to be promising. Consequently, the acquisition of the right amount of amplifiable DNA for obtaining reliable data when the amount of input DNA is fairly low should be taken into account.

On the other hand, most kits except the T-EJ showed relatively large variances in recovery, which could affect the reliability in forensic DNA methylation works when the amount of BS-DNA is much lower than the analytical limit for an accurate and reliable DNA methylation study. Consequently, the recovery should be considered high on the list when there is a limitation on the amount of available DNA. Also, when it comes to the study of a small amount of DNA, it is possible to obtain more reproducible and reliable DNAm data by using a normalized amount of BS-DNA based on the BisQuE result.

## Conclusion

In this study, the BisQuE system for both gDNA and BS-DNA was developed. It is a simple single assay to measure the amount of both gDNA and BS-DNA by calculating the Ct values of short-C, short-T, and long-Cfree. Cfree primers used in this method enabled the amplification of DNA regardless of whether it is BS-converted or not. Inspired from the commercially available kit, the BisQuE targeted two different-sized amplicons for degradation level during the BS conversion steps. Also, the transforming equation of short-T to short-C, which was obtained from the C-T indicator, was adopted to compute the amount of the converted DNA. Based on the amount of measured DNA, three key features of the conversion step (BS conversion efficiency, the degradation level, and the recovery) were calculated. Exploiting the constructed qPCR system, six BS conversion kits were tested in 20 samples using 50 ng of gDNA as an input. A total of 99% conversion efficiency was shown by most kits, whereas the other kit was about 94%. The enzymatic modifying N-NE kit showed the lowest degradation level, whereas other kits similarly ranged between 1.279 and 1.762. Kit recovery was also investigated and found to range between 18% and 50%. DNA recovery should be considered thoroughly when the amount of the available DNA samples is limited. Furthermore, the amount of amplifiable DNA cannot be guaranteed by the converted DNA either, even though the results of the Qubit seem to be promising. For other studies, it is highly recommended to determine the BS conversion kit according to the purpose of the specific study. Moreover, BS-DNA input should be considered before the DNAm analysis to guarantee the accuracy of downstream analysis.

## Data Availability Statement

The original contributions presented in the study are included in the article/Supplementary Material, further inquiries can be directed to the corresponding author/s.

## Ethics Statement

The studies involving human participants were reviewed and approved by the Institutional Review Board of Severance Hospital, Yonsei University in Seoul, South Korea (4-2019-0707). The patients/participants provided their written informed consent to participate in this study.

## Author Contributions

K-JS and SH: study conception. K-JS: study supervision and obtained funding. SH: conducting analyses and data interpretation and writing manuscript. Both authors contributed to the manuscript revision, read, and approved the submitted version.

## Conflict of Interest

The authors declare that the research was conducted in the absence of any commercial or financial relationships that could be construed as a potential conflict of interest.

## Footnotes

1 http://bioinfo.ut.ee/primer3-0.4.0/

2 https://faculty.ucr.edu/~mmaduro/random.htm
