# Supplementary figures and images for: Bisulfite-Converted DNA Quantity Evaluation: A Multiplex Quantitative Real-Time PCR System for Evaluation of Bisulfite Conversion
Source: Front Genet. 2021 Feb 25;12:618955. doi: 10.3389/fgene.2021.618955 (PMC7947210; doi:10.3389/fgene.2021.618955)

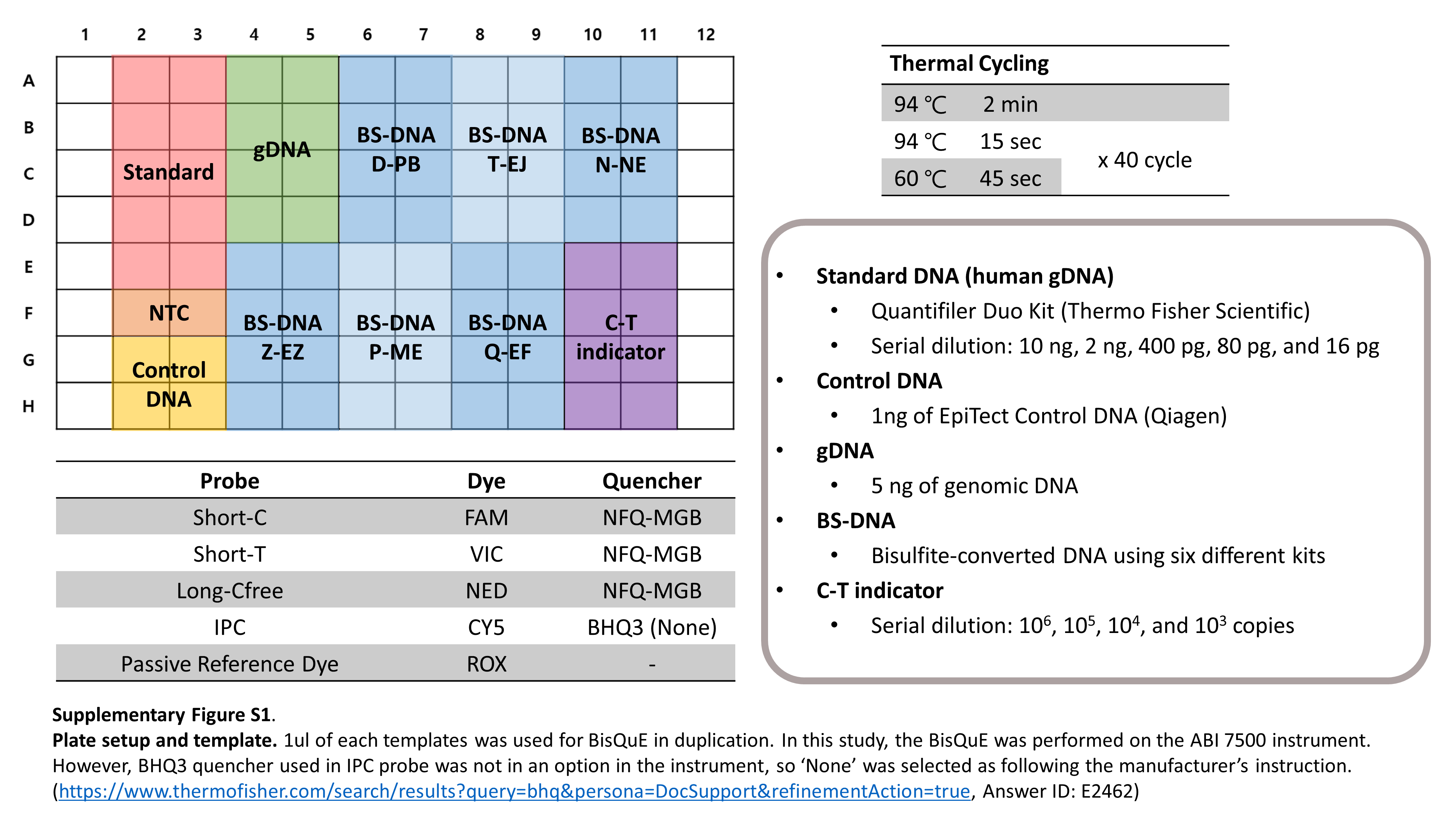

Supplement: Supplementary file 1 [file Image_1.TIF]

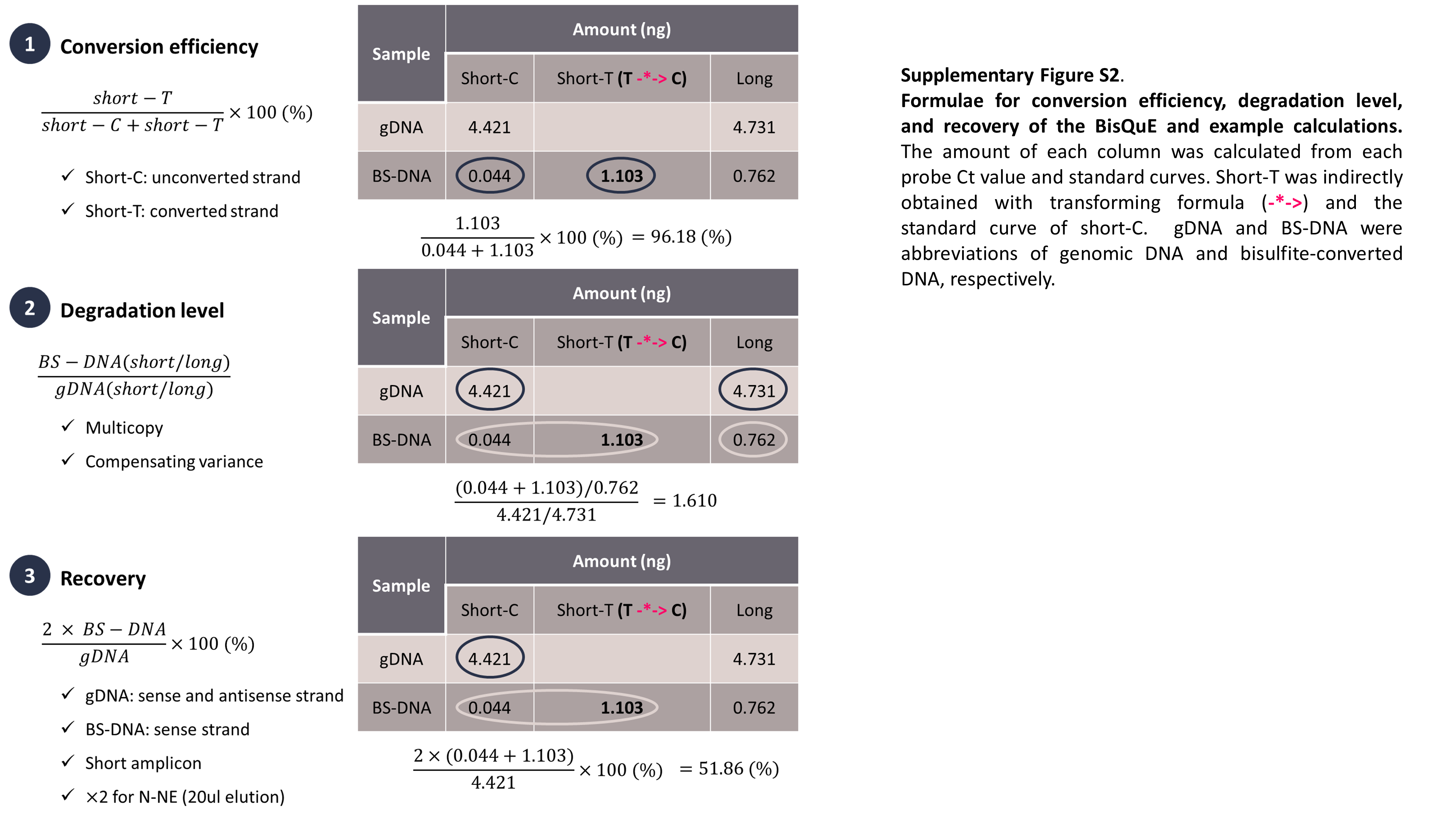

Supplement: Supplementary file 2 [file Image_2.TIF]

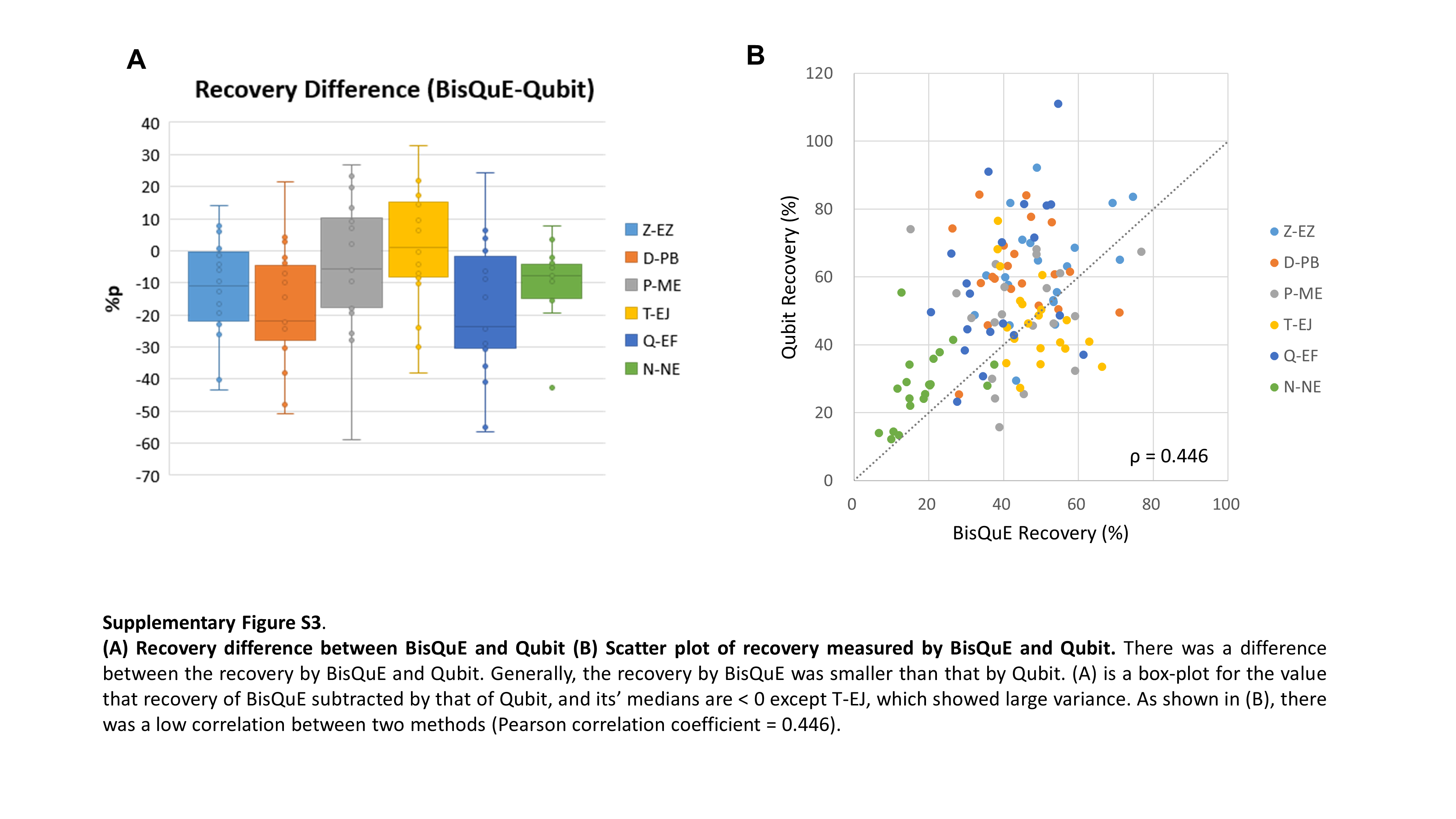

Supplement: Supplementary file 3 [file Image_3.TIF]

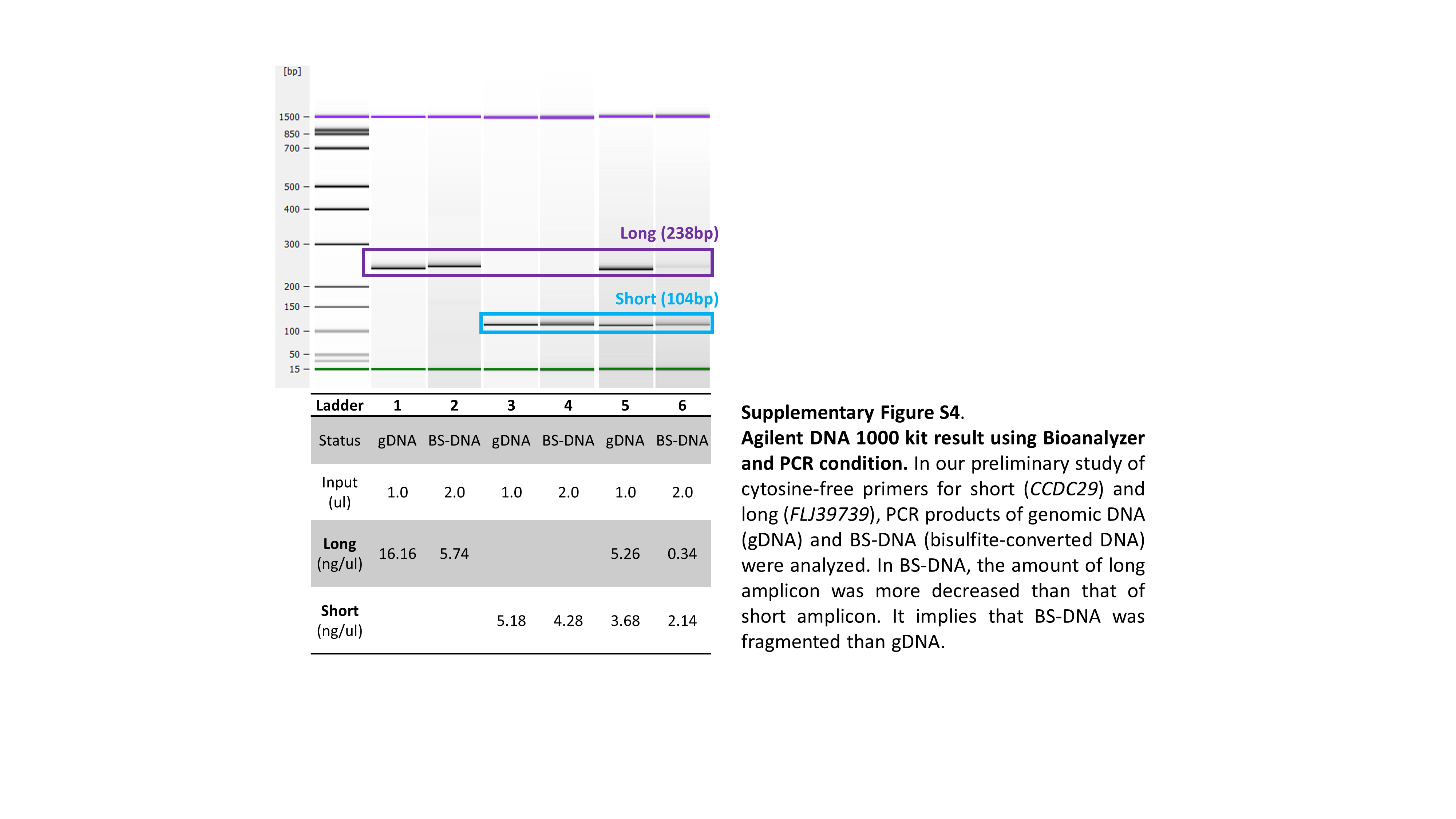

Supplement: Supplementary file 4 [file Image_4.TIF]
