# Supplementary material for: Bisulfite-Converted DNA Quantity Evaluation: A Multiplex Quantitative Real-Time PCR System for Evaluation of Bisulfite Conversion
Source: Front Genet. 2021 Feb 25;12:618955. doi: 10.3389/fgene.2021.618955 (PMC7947210; doi:10.3389/fgene.2021.618955)
Supplement: Supplementary file 5 [file Table_1.DOCX]

**Table S1.** Six BS conversion kits and its’ instructions.

| No. | Manufacturer | Kit | DNA Input | Input vol. (µl) | Elution vol. (µl) |
| --- | --- | --- | --- | --- | --- |
| 1 | Zymo Research | EZ DNA Methlyation-Lightning Kit | 100 pg - 2 μg | 20 | 10 |
| 2 | Diagenode | Premium Bisulfite kit | 100 pg - 2 μg | 20 | 10 |
| 3 | Promega | MethylEdge Bisulfite Conversion System | 100 pg - 2 μg | 20 | 10-20 |
| 4 | Thermo Fisher Scientific | EpiJET Bisulfite Conversion Kit | 50 pg - 2 μg | 20 | 10-20 |
| 5 | Qiagen | EpiTect Fast Bisulfite kit | 1 ng – 2 µg | 40 | 10-15 |
| 6 | New England Biolabs | NEBNext Enzymatic Methyl-seq Conversion Module | 10 ng - 200 ng | 29 | 20 |
