# Supplementary material for: Bisulfite-Converted DNA Quantity Evaluation: A Multiplex Quantitative Real-Time PCR System for Evaluation of Bisulfite Conversion
Source: Front Genet. 2021 Feb 25;12:618955. doi: 10.3389/fgene.2021.618955 (PMC7947210; doi:10.3389/fgene.2021.618955)
Supplement: Supplementary file 6 [file Table_2.DOCX]

| **C-T indicator**  **(single strand)**  **104 nt** | GAA ATG GTT AAG AGA AAG GGA AAA ACT GAA ACC TGT GGG TGA ATA **Y**TT AGA ATG ACA GTA TTT AGC TCA GCC TGA AGA CAG ATG AGG ATG AAA AAT GTA ATG GG |
| --- | --- |
| **IPC**  **(dsDNA)**  **450 bp** | CTCTAACTAGTATGGATAACCGTGTTTTCACTGTGCTGCGGTTACCCATCGCCTGAAATCCAGTTGGTGTCAAGCCATTCCCTGTCTAGGACGCCGCATGTAGTAAAACATATACATTGCTCGGGTTCACTCCGGTCCGTTCTGAGTCGACCAAGGACACAATCGAGCTCCGATCTGTATTGTCGAGAAACTTGTATCCAACCCCCGCAGCTTGCCAGCTCTTCGGGTATCATGGAGCCTATGGTTGAACAAGGCCCATACGCGAGATAAACTGCTAGAAAACCGCGTCTTTACGACTGGTGCTTAATTTAATTTCGCTGACGTGATGACATTCCAGGCAGTGCGTCTGCTGTCGGGTCCCTCTCGTGATTGGGTAGTTGGACATGCCCTTGAAAAACATAGCAAGAGCCTGCCTCTCTATTGATGTCACGGCGAATGTCGGGGAGACAG |

**Table S2**. C-T indicator, and IPC sequence for real-time qPCR.
