# Supplementary material for: Bisulfite-Converted DNA Quantity Evaluation: A Multiplex Quantitative Real-Time PCR System for Evaluation of Bisulfite Conversion
Source: Front Genet. 2021 Feb 25;12:618955. doi: 10.3389/fgene.2021.618955 (PMC7947210; doi:10.3389/fgene.2021.618955)
Supplement: Supplementary file 7 [file Table_3.DOCX]

**Table S3.** Standard curve and brief Ct value of Standard DNA and in five real-time PCR assays.

|  | Short-C | | | | | Long-Cfree | | | |
| --- | --- | --- | --- | --- | --- | --- | --- | --- | --- |
| Standard DNA (ng/well) | Min | Max | Average | SD* | Min | | Max | Average | SD* |
| 10 | 21.034 | 21.427 | 21.247 | 0.145 | 22.479 | | 22.738 | 22.595 | 0.083 |
| 2 | 23.209 | 23.697 | 23.510 | 0.173 | 24.728 | | 24.953 | 24.850 | 0.071 |
| 0.4 | 25.390 | 25.975 | 25.742 | 0.218 | 26.990 | | 27.140 | 27.059 | 0.061 |
| 0.08 | 27.662 | 28.143 | 27.932 | 0.176 | 28.897 | | 29.408 | 29.182 | 0.146 |
| 0.016 | 29.423 | 30.167 | 29.899 | 0.240 | 30.838 | | 31.350 | 31.077 | 0.157 |
| Assay | Slope** | Y-intercept | R-squared | PCR Efficiency (%) | Slope** | | Y-intercept | R-squared | PCR Efficiency (%) |
| 1 | -2.2005 | 24.458 | 0.9985 | 107.80 | -2.1478 | | 25.812 | 0.9982 | 111.56 |
| 2 | -2.2141 | 24.511 | 0.9983 | 106.87 | -2.1241 | | 25.676 | 0.9989 | 113.34 |
| 3 | -2.1606 | 24.493 | 0.9992 | 110.62 | -2.1429 | | 25.704 | 0.9989 | 111.92 |
| 4 | -2.1235 | 24.513 | 0.9975 | 113.38 | -2.1185 | | 25.742 | 0.9986 | 113.76 |
| 5 | -2.1642 | 24.170 | 0.9996 | 110.36 | -2.1150 | | 25.767 | 0.9992 | 114.03 |

*SD denotes of standard deviation.

**log 5 scale
