# Supplementary material for: Bisulfite-Converted DNA Quantity Evaluation: A Multiplex Quantitative Real-Time PCR System for Evaluation of Bisulfite Conversion
Source: Front Genet. 2021 Feb 25;12:618955. doi: 10.3389/fgene.2021.618955 (PMC7947210; doi:10.3389/fgene.2021.618955)
Supplement: Supplementary file 8 [file Table_4.DOCX]

**Table S4.** Short-T to short-C Ct transforming equations using C-T indicator of five real-time PCR assays.

|  | Short-C | | | | Short-T | | | |
| --- | --- | --- | --- | --- | --- | --- | --- | --- |
| C-T indicator copy numbers | Min | Max | Average | SD* | Min | Max | Average | SD* |
| 10^6^ | 23.853 | 23.360 | 23.093 | 0.154 | 24.228 | 24.936 | 24.600 | 0.262 |
| 10^5^ | 26.182 | 26.395 | 26.292 | 0.086 | 27.447 | 28.211 | 27.786 | 0.231 |
| 10^4^ | 29.209 | 29.645 | 29.414 | 0.146 | 30.464 | 31.235 | 30.796 | 0.276 |
| 10^3^ | 31.586 | 32.268 | 31.974 | 0.241 | 32.903 | 34.492 | 33.637 | 0.505 |
| Assay | Slope | Y-intercept | R-squared |  |  |  |  |  |
| 1 | 0.9719 | -0.4951 | 0.9962 |  |  |  |  |  |
| 2 | 0.9700 | -0.4548 | 0.9991 |  |  |  |  |  |
| 3 | 1.0092 | -1.9415 | 0.9996 |  |  |  |  |  |
| 4 | 0.9891 | -1.5536 | 0.9988 |  |  |  |  |  |
| 5 | 1.0033 | -1.4724 | 0.9997 |  |  |  |  |  |

*SD denotes of standard deviation.
