# Supplementary material for: Bisulfite-Converted DNA Quantity Evaluation: A Multiplex Quantitative Real-Time PCR System for Evaluation of Bisulfite Conversion
Source: Front Genet. 2021 Feb 25;12:618955. doi: 10.3389/fgene.2021.618955 (PMC7947210; doi:10.3389/fgene.2021.618955)
Supplement: Supplementary file 9 [file Table_5.DOCX]

**Table S5.** Internal positive control Ct values of five real-time PCR assays.

| Assay | Genomic DNA | | | | Bisulfite converted DNA | | | |
| --- | --- | --- | --- | --- | --- | --- | --- | --- |
|  | Min | Max | Average | SD* | Min | Max | Average | SD* |
| 1 | 26.681 | 28.001 | 27.339 | 0.395 | 27.083 | 27.800 | 27.408 | 0.188 |
| 2 | 27.028 | 28.275 | 27.517 | 0.387 | 27.212 | 28.031 | 27.646 | 0.218 |
| 3 | 27.075 | 28.307 | 27.465 | 0.371 | 27.178 | 27.976 | 27.541 | 0.178 |
| 4 | 26.961 | 28.194 | 27.401 | 0.406 | 27.088 | 28.047 | 27.435 | 0.189 |
| 5 | 27.013 | 28.297 | 27.568 | 0.321 | 27.233 | 27.972 | 27.561 | 0.161 |

*SD denotes of standard deviation
